# Risks of dioxins resulting from high exposure via breast-feeding?

**DOI:** 10.1007/s00204-017-1952-7

**Published:** 2017-03-18

**Authors:** Klaus Abraham

**Affiliations:** Department of Food Safety, German Federal Institute for Risk Assessment (BfR), Max-Dohrn-Str. 8-10, 10589 Berlin, Germany

Commentary to,

Martin van den Berg, Karin Kypke, Alexander Kotz, Angelika Tritscher, Seoung Yong Lee, Katarina Magulova, Heidelore Fiedler & Rainer Malisch.

WHO/UNEP global surveys of PCDDs, PCDFs, PCBs and DDTs in human milk and benefit-risk evaluation of breastfeeding. Archives of Toxicology, January 2017, Volume 91, Issue 1, pp 83–96.

As part of the article by Martin van den Berg and others, the authors compare possible risks and benefits of breast-feeding in relation to the levels of PCDDs and PCDFs (“dioxins”) and other persistent compounds. For dioxins, a “safe level” of 0.2 pg WHO-TEQ per g fat of mother’s milk was calculated, using the lower WHO-TDI of 1 pg/kg b.w., a fat content of 3.5% and a daily consumption of 125 g/kg b.w. This is a simple and correct calculation—if it makes sense to apply the TDI to breastfed infants in this way.

For dioxins, the TDI also considers the extremely long biological half-life of the compounds in order to prevent a critical body burden in the child-bearing age (females) or in aged people possibly associated with health effects. Therefore, a slight and temporary exceedance of the TDI is acceptable, but what about breast-feeding? What about dioxin body burden of infants who are exclusively breastfed for up to 6 months, taking up, for example, 44 pg/kg b.w. per day during this period in case of a maternal body burden of 10 pg/g fat (using the same numbers for the calculation as above)? At first glance, such a calculation gives rise to a concern regarding a critical body burden, primarily for females in the child-bearing age.

Kinetic models predict the expected big difference in dioxin body burden between formula-fed and breast-fed infants at the end of the breast-feeding period (Kreuzer et al. 1997; Kerger et al. 2007). However, concentrations in the former breast-fed child decline rapidly thereafter, due to the fast growth of body mass and body fat, and an age-dependent elimination half-life (Kerger et al. 2006) may also contribute to this process. Within a few years, this leads to an assimilation of the child’s dioxin concentrations to the slowly increasing concentrations of a child who has never been breastfed. These model predictions were validated by measured data. Therefore, former breast- and formula-fed girls living under otherwise identical conditions are not expected to differ with respect to their body burden in the child-bearing age, as long as the contamination of mother’s milk does not greatly exceed the current background range (WHO-TEQ <20 pg/g fat). In conclusion, no increased risk results from breast-feeding of girls for their future offspring during the in-utero period.

Another question is, whether the temporarily (up to a few years) higher dioxin body burden in breast-fed children during a period of possibly higher susceptibility is associated with health risks. Indeed, infants exclusively breastfed for 6 months according to current recommendations, reached dioxin levels comparable to or even slightly higher than those of their mothers (Abraham et al. 1996, 1998). Numerous studies have focused on the question of possible health risks or changes in laboratory parameters in children breastfed for a longer period. Currently, there is no serious evidence for any of that. Even after the Seveso accident in 1976, no health effects have been reported for children primarily exposed via breast-feeding.

Dioxin levels in human milk are strongly decreasing in industrialized countries—in Germany from mean values of about 35 pg WHO-TEQ pg per g milk fat at the end of the 1980s to 6.3 WHO-TEQ pg per g milk fat in 2009 (BfR 2011; more recent data not available). These numbers are 175 and 32 times higher than the proposed “safe level” of 0.2 pg WHO-TEQ per g milk fat, respectively. Scientifically, establishing such a “safe level” with these dramatic exceedances without serious evidence for negative health effects appears questionable.

Of course, dioxins are unwanted contaminants and should be further reduced with special emphasis on possible in-utero effects. However, from the pediatrician’s view on the current dioxin levels in human milk from industrialized countries (<20 pg WHO-TEQ per g fat), it makes no sense to point out putative dioxin risks of breast-feeding over and over again, even if the final conclusion is that the benefits are higher than the risks. This may contribute to unnecessary confusion of parents and health professionals, and thereby bears the risk of negatively influencing breast-feeding rates.
